# 

**Published:** 1910-05-07

**Authors:** 


Alcohol should be given with the same strict dosage and regularity as medicine, and it should not be forgotten that stimulants, when really required, are most necessary during the night. It is sometimes advisable to prescribe alcohol in such a form as the following: Tr. Aurantii ʒii., Sp. Ætheris ʒss., Sp. Ammon. Aromat. ʒss., Tr. Nucis Vom. ♏x., Aquam ad ℥j.—*Dr. Essex Wynter.*

In January 1908 the Research Defence Society was founded. Its objects are to make generally known the facts about experiments on animals in this country and the regulations under which they are conducted, the immense importance of such experiments to the welfare of mankind, and the great saving of human and animal life and health which is already due to them. With these ends in view, the society gives information to all who wish to examine the arguments on behalf of experiments on animals, makes arrangements for addresses and lantern lectures, and publishes and distributes literature. It already has a membership of more than 3,000. It has been felt desirable by the members of the South-West London Medical Society and the members of the Wandsworth Division of the British Medical Association that a branch of the Research Defence Society should be formed in the South-Western District of London. A joint meeting of the Councils of the two societies has been held and a provisional committee appointed, with Dr. Biggs as President (*pro tem.*) and Dr. C. Melville Young as Hon. Secretary (*pro tem.*). The subscription for membership is 5s., or for associateship 1s. per annum.

### THE GRADUATES' MEDICAL SCHOOLS.

DIARY FOR THE WEEK, MAY 9 to MAY 14.

**THE POST-GRADUATE COLLEGE,** West London Hospital, Hammersmith, W.

At 10 a.m.—**May 9 and 12,** Demonstrations by Surgical Registrar.
10 a.m.—**May 13,** Demonstration by Medical Registrar.
10.30 a.m.—**May 10,** Demonstrations of Minor Operations.
12.15 p.m.—**May 11,** Dr. Grainger Stewart, **Practical Medicine.**
5 p.m.—**May 9,** Dr. Davis, **Diseases of the Throat, Nose, and Ear.**
5 p.m.—**May 10,** Dr. Seymour Taylor, **Acute Rheumatism.**
5 p.m.—**May 11,** Dr. Beddard, **Practical Medicine** (Lecture I.).
5 p.m.—**May 12,** Dr. Davis, **Diseases of the Throat, Nose, and Ear.**
5 p.m.—**May 13,** Dr. Abraham, **Cases of Skin Disease.**

**CENTRAL LONDON THROAT AND EAR HOSPITAL,** Gray's Inn Road, W.C.

At 3.45 p.m.—**May 10,** Mr. J. G. French, **Tracheoscopy.**

**LONDON SCHOOL OF CLINICAL MEDICINE,** Seamen's Hospital, Greenwich, S.E.

At 4 p.m.—**May 9,** Mr. R. Lake, **Nasal Obstruction.**
2.15 p.m.—**May 11,** Dr. F. Taylor, **Empyema.**
4 p.m.—**May 12,** Dr. Hawthorne, **Some Aspects of Rheumatism in Early Life.**

**ROYAL SOCIETY OF MEDICINE,** 15 Cavendish Square, W. (Temporary address during building.)

At 5.30 p.m.—**May 9, Medical Section:**
At 8.30 p.m.—**May 9,** Sir P. Manson, **Malaria.** Dr. F. M. Sandwith, **Ankylostomum Duodenale.** Dr. Carnegie Brown, **Tropical Dysentery.**
Annual General Meeting : Election of officers and council for session 1910-1911.
At 5.30 p.m.—**May 10, Surgical Section:**
At the Botanical Theatre, University College, W.C.
*Discussion* on "The present position of the treatment of syphilis." (To be continued at the next meeting on June 14.) Mr. Ernest Lane will open the discussion, and Sir Jonathan Hutchinson, Dr. Alexander Fleming, Mr. Arthur Shillitoe, Mr. H. W. Bayly, Mr. J. E. R. McDonagh, Dr. F. J. Smith, Mr. C. F. Marshall, Dr. G. Pernet, Mr. Charles Gibbs, and Captain Rennie will take part.
At 7.45 p.m.—**May 12, Obstetrical and Gynæcological Section:**
Annual General Meeting : Election of officers and council.
*Papers* : Dr. F. J. McCann : "Cæsarean Section in the Treatment of Eclampsia Gravidarum." Dr. A. J. Wallace : "Contribution to the Life-history of Fibromyomata of the Uterus."
At 5 p.m.—**May 13, General Meeting of Fellows:** Election of Candidates for Fellowship.
At 8.30 p.m.—**Clinical Section:**
Annual General Meeting : Election of officers and council. Dr. Henry Head : Demonstration on "Clinical Methods for Testing Sensation"—illustrated by cases. Dr. R. G. Hebb : Lymphadenoma with pruritus and cutaneous tumours. Dr. A. C. Firth : A case of pink urine caused by eating highly coloured sweets. Dr. R. C. Elmslie : (1) Three cases of enlargement of the tubercle of the tibia (Schlatter's disease). (2) A case of fracture of the humerus at the site of an innocent cyst. And other cases.
ANNOUNCEMENT.—Special Meeting of Fellows of the Society : May 18, at 5 p.m., at Morley Hall, George Street, Hanover Square, W. Sir Almroth Wright, F.R.S., will open a discussion on "Vaccine Therapy : Its Administration, Value, and Limitations." The discussion will be continued on May 25, June 1, June 8, and June 15. The Council has arranged for a number of Fellows to speak on the above days. Any Fellow who wishes to take part in the discussion is requested to communicate with the hon. secretaries.

**NATIONAL HOSPITAL FOR THE PARALYSED AND EPILEPTIC,** Queen Square, Bloomsbury, W.C.

At 3.30 p.m.—**May 10,** Dr. Gordon Holmes, **Functional Localisation in the Brain.**
**May 13,** Dr. Ormerod, **Tabes Dorsalis.**

**MEDICAL GRADUATES' COLLEGE AND POLYCLINIC,** 22 Chenies Street, W.C.

At 4 p.m.—**May 9,** Dr. George Pernet, **Clinique (skin).**
5.15 p.m.—**May 9,** Dr. Thomas Eden, **Some Common Difficulties in Forceps Delivery.**
4 p.m.—**May 10,** Dr. Purves Stewart, **Clinique (medical).**
5.15 p.m.—**May 10,** Dr. T. Claye Shaw, **Suggestion, Hypnotism, and Telepathy.**
4 p.m.—**May 11,** Mr. James Cantlie, **Clinique (surgical).**
5.15 p.m.—**May 11,** Mr. J. Bland-Sutton, **Injuries of the Heart.**
4 p.m.—**May 12,** Dr. James Mackenzie, **Clinique (medical).**
5.15 p.m.—**May 12,** Mr. Jackson Clarke, **Internal Derangements of the Knee-joint.**
4 p.m.—**May 13,** Dr. William Hill, **Clinique (Ear, Nose, and Throat).**

Sir Henry Grey has been re-elected President of the Malvern Hospital. Other re-elections were Mr. F. Lloyd Oswell, Hon. Treasurer; Mr. A. H. Weller, Secretary; and Mr. W. Higley, Hon. Auditor. The following Committee, of which Sir Henry Grey acts as Chairman, was also re-elected: The Rev. F. W. Davenport, the Rev. A. C. Deane, the Rev. Walter Lee, and the Rev. H. Maynard Smith, Major Hill, Mr. W. D. Perrins, Mr. G. Ellis, Mr. R. A. Hollins, Mr. T. Kensington, Mr. J. A. Meyer, Mr. W. Vernall, and Mr. W. Walton.

At the half-yearly meeting of the Governors of the Derbyshire Royal Infirmary, over which Sir Arthur Heywood presided, the following governors were chosen to serve on the Special Elective Committee for three years: Mr. G. B. Barrington, Kirk Langley, Derby; Mr. Alfred Clay, Darley Hall, Matlock; Sir Thomas Roe, M.P., Grove Villas, Derby; Mr. E. M. Longdon; Mr. H. Brooke Taylor, The Hall, Bakewell; Mr. W. Gladwyn Turbutt, Ogston Hall, Alfreton; Mr. J. G. Wilson, The Firs, Alfreton; and Mr. G. H. Wheatcroft, Waltham House, Wirksworth, Derby. It was the Special Elective Committee which appointed the Rev. Jonathan Howell to be chaplain to the Infirmary earlier in the year.

### THE QUESTION BOX.

**SPECIAL NOTICE.**—Questions of interest affecting the honorary medical staff and hospital administration in all departments are invited. When any point arises we hope our readers will formulate it in a question and so co-operate to make this column of real value and interest. In this way the experience of the best-managed hospitals should be made helpful to the whole hospital world. We shall welcome the contribution of both questions and answers.

**N.B.**—The publication of an answer in this column does not necessarily preclude the publication of subsequent answers to the same question, for a question may involve points which require to be threshed out and fully discussed. Answers, if published, will be paid for at scale rates.

### HOSPITAL ADMINISTRATION.

Question: Is it desirable that a chair of hospital administration be established at one or more of the Universities?

Answer: Not at present.

By a Medical Superintendent.

If Huxley's characteristic saying that "The student to whose wants the mediæval University was adjusted looked to the past and sought book-learning, while the modern looks to the future and seeks knowledge of things" be true, then no doubt there is something to be said for the establishment of a University chair of hospital administration. But what occurs to me as the chief objection to this is that the problem of problems in hospital administration is the very unacademical pursuit of money-getting. It is a fact that as things are a "pushful" man who has the ear of people in the City is of much more use to a charitable institution than one ever so highly trained in account keeping and household management. But if things progress as they look like doing, if co-operation and amalgamation on a large scale come to pass, if we get hospital farms and central stores and municipal oversight, then the day of the commonsense man who gets hold of good subordinates and trusts to what they tell him will come to an end. Perhaps other of your correspondents have a more definite opinion to give; mine must be expressed in this conditional mood.

### Gifts and Bequests.

Lord Airedale has given £100 to the Convalescent Home for men at Horsforth, near Leeds.

Mr. George Norman Maule, of Ilfracombe, Devon, has left £300 to the Tyrrell Cottage Hospital, Ilfracombe.

Intimation has been received by the Leicester Infirmary of a bequest of £100 free of duty from Mr. Henry Jackson, of Melton Mowbray.

The hon. secretaries of King Edward's Hospital Fund for London have received at the Bank of England, from the executors of the late Mr. Richard Clarke, the sum of £4,919 10s. 6d., being the amount of the residuary estate bequeathed by the deceased to the fund.

The treasurer of University College Hospital has received the following donations in response to the special appeal for £10,000 before June 1 next: Mr. Claude G. Montefiore, £200; Sir Thomas Barlow and Lady Barlow, each £100; Sir Peter Spokes, £52 10s.; Mr. Hugh Martineau, Mr. Arthur Lucas, Mrs. L. Hardy, "A. W.," Mrs. Montague Ballard, Lady Wantage, Lady Theodora Guest, and "Anon.," each £50; Mr. Walter Baily, Mrs. Walter Baily, and Mr. C. Fellows Pearson, each £30; Lord Tavistock, Sir Augustus Prevost, and Mr. Richard Worsley, each £25; and Mr. I. S. Lister, £20.

The Duke of Devonshire has promised to give £500 towards the expense of enlarging Princess Alice's Hospital, Eastbourne. Mr. Sydney Hudson has given £500 also.

Lord Sandhurst, treasurer of St. Bartholomew's Hospital, has received from the Corporation of London £200, a further instalment of their donation to the rebuilding fund.

Mr. Sigismund Loewen Helm, of Crumpsall, Manchester, formerly shipping merchant, has left subject to various bequests the residue of his estate for the following charitable purposes in Manchester: Five-twentieths to the Northern Counties Hospital for Incurables, three-twentieths each to the David Lewis Manchester Colony of Epileptics, Ancoats Hospital, and the Cancer Pavilion and Home, and two-twentieths each to the Victoria Dental Hospital, the Hospital for Consumption and Diseases of the Throat and Chest, and the Clinical Hospital for Women and Children.

### Jottings.

The Princess of Wales has sent a hamper of primroses for the decoration of the wards of the Great Northern Central Hospital.

A meeting was held on Monday to consider the scheme for enlarging Princess Alice's Hospital, Eastbourne. The Duke of Devonshire was in the chair.

Lord Strathcona will preside at the Festival Dinner in aid of the Metropolitan Hospital, Kingsland Road, to be held at the Whitehall Rooms on Monday, June 20.

The Garrick Amateur Dramatic Society gave "The Merchant of Venice," in Sir Henry Irving's version, at the Court Theatre at 8 p.m. yesterday (and repeat it to-day) in aid of the French Hospital.

The annual general meeting of the West London Hospital Ladies' Association was held last Friday at 3.30 p.m., when the Vice-President, Mary Lady Ilchester, presided.

The Sixty-fifth Anniversary Dinner of the German Hospital, Dalston, will take place at the Whitehall Rooms, Hôtel Métropole, on Thursday, May 19, when the Right Hon. Sir Frank Cavendish Lascelles, G.C.B. G.C.M.G., G.C.V.O. will preside.

Princess Alexander of Teck and Mrs. Alfred Mond have arranged the programme of the special matinée in aid of the Royal Waterloo Hospital for Children and Women and the Infants' Hospital, to be given at His Majesty's Theatre on May 23. At the request of Princess Alexander, Mrs. Henry de la Pasture has dramatised "The Unlucky Family," her Christmas story for children. Miss Rosini Filippi and Miss Viola Tree will produce the play under the personal direction of the author, while Miss Margaret Morris will arrange the dances. The second part of the programme will include a *Soirée Watteau.*

The charity matinée on behalf of the Charing Cross Hospital, to take place at the Adelphi on Thursday, June 23, is to be Japanese in character, and, instead of a long succession of turns, three plays are to be given—namely, "The Mirror," founded on a Japanese legend by Rosina Filippi; "A Japanese Revenge," dramatised from a story by Sir Arthur Conan Doyle; and "Jappy Chappy," a children's musical extravaganza. The plays are being produced by Miss Italia Conti, with a committee of ladies, including Lady Lytton, Lady Juliet Duff, Lady Paget, Mrs. Ronalds, and Mrs. Schuster. The names of the artists taking part will be announced shortly.

Mr. R. Ford has been elected Chairman of the Executive Committee of the Malton Workpeople's Hospital Fund, of which the other officers are: Vice-Chairman, Mr. Robert Wilson; Treasurer, Mr. J. Rudsdale; Secretary, Mr. C. Glenday; Assistant Secretary, Mr. Geo. Barker. The workpeople's representative on the hospital board will be an *ex-officio* member of the fund committees.

Mrs. Senhouse has been re-elected President of the Maryport Cottage Hospital, and the committee has been chosen as follows: Co-operative Society's President, Mr. Burnett (Chairman of the Urban District Council), the Rev. J. A. Richards, Father Polding, Mr. A. Crerar, Mr. J. Hannah, Mr. Waite, Mr. A. Gibb, and Mr. T. Blain. Ladies' Visiting Committee: Mrs. Proud, Mrs. Bowie, Mrs. Leonard, Mrs. Gibb, Mrs. Maughan, Mrs. Glaister, Mrs. Blain, Mrs. G. Ferguson, Mrs. Kennedy, Mrs. Clark, Mrs. Richards, and Mrs. Crerar.

### COMING EVENTS OF THE WEEK.

**Saturday, May 7.**—Carnival at Bramley in aid of Leeds Hospital. Municipal and Health Exhibition opens, Royal Agricultural Hall, Islington, N. (eight days).

**Sunday, May 8.**—Visiting Day at all hospitals.

**Monday, May 9.**—Medical Society: Annual Meeting, 8; Ordinary, 8.30.

Chemists' Exhibition, Royal Agricultural Hall (five days).

**Tuesday, May 10.**—Duchess of Albany at National League for Physical Education and Improvement, at 6 Gloucester Terrace, Hyde Park, 3.30 p.m.

Meeting in support of fund for London University New Chemical Laboratories, Lord Rosebery and others, Mansion House, 4 p.m.

**Wednesday, May 11.**—Annual Dinner Royal Hospital for Incurables, Putney, Grocers' Hall, Princes Street (?), E.C. 6.30.

League of Mercy, N.E. Sussex District: the Hon. Mrs. Marshall "At Home," Cranesden, Mayfield, 3 p.m.

**Thursday, May 12.**—Festival Dinner City of London Hospital for Diseases of Chest. Hon. Walter Rothschild presiding, Trocadero, 6.30.

League of Mercy, N.E. Sussex District: A meeting will be held at Carpenter's Restaurant, 3 p.m., organised by Mrs. Huth.

### THE MELBOURNE HOSPITALS.

All the Melbourne hospitals at present are in process of rearrangement. The Melbourne, which is the largest general hospital, owing to a falling-off in subscriptions and a continuous rise in the price of provisions, has a total deficit of £4,000. The Hospital for Infectious Diseases is seeking to increase its funds by compelling all municipalities to contribute a fixed sum. The Women's Hospital is making public appeal for help, and is also in urgent need of more infirmary accommodation, as there are sometimes over 200 women waiting for admission. Private hospitals are being brought to registration every year, this formality often being allowed to lapse after the first year. The need of "intermediate" hospitals for the less well-off classes, who yet do not desire free medical assistance, having become painfully apparent from the misuse of the public charitable institutions, a proposal that the State should establish them has been made. Fortunately this idea has not been entertained by the Government, and has been left to private enterprise to carry out. After long discussion, the Melbourne Hospital has set about reforming its method of electing its honorary physicians and surgeons, which has been done hitherto by the subscribers and governors. An electoral board is likely to be appointed, which will still leave much power with the committee.

